# Implementation of uterine artery embolization for symptomatic fibroids in the Netherlands: an inventory and preference study

**DOI:** 10.1186/s42155-019-0061-5

**Published:** 2019-06-03

**Authors:** Annefleur M. de Bruijn, Jolijn Huisman, Wouter J. K. Hehenkamp, Paul N. M. Lohle, Jim A. Reekers, Anne Timmermans, Andries R. H. Twijnstra

**Affiliations:** 1 Department of Gynecology, Amsterdam University Medical Center, Vrije Universiteit Amsterdam, De Boelelaan 1117, 1007 MB Amsterdam, The Netherlands; 2 Faculty of Medicine, VU University, De Boelelaan 1105, 1081 HV Amsterdam, The Netherlands; 3 Department of Radiology, Elisabeth Tweesteden Ziekenhuis, Tilburg, The Netherlands; 4 Department of Radiology, Amsterdam University Medical Center, University of Amsterdam, Meibergdreef 9, Amsterdam, the Netherlands; 5 Department of Obstetrics and Gynecology, Amsterdam University Medical Center, University of Amsterdam, Meibergdreef 9, Amsterdam, The Netherlands; 6 Leiden University Medical Center, Albinusdreef 2, 2300-2333 ZA Leiden, the Netherlands

**Keywords:** Uterine artery embolization, Heavy menstrual bleeding, Hysterectomy, Annual numbers, Counselling, Consent

## Abstract

**Background and purpose:**

The Dutch national guideline on heavy menstrual bleeding was updated and published in 2013. It recommended (for the first time) that uterine artery embolization (UAE) should be part of counseling of women with symptomatic fibroids. We aimed to evaluate the implementation of UAE for symptomatic uterine fibroids in the Netherlands and to investigate gynecologists preference and other influential factors.

**Methods:**

The primary outcome was to examine the UAE/hysterectomy ratio before and after introduction of the 2013 guideline by the use of annual hospital reports. The secondary outcome assessed factors that could influence implementation by means of a questionnaire to gynecologists.

**Results:**

A total of 29/30 (97%) UAE+ hospitals and 36/52 (69%) UAE- hospitals sent their annual reports. The UAE/hysterectomy percentages in 2012, 2013 and 2014 were 7,0%, 7.0% and 6.9%, respectively. Regarding the questionnaire, the response rates were 88% and 91%, respectively. In both groups we observed a high self-perceived tendency for UAE counseling (90% versus 70%, *p* = .001). Approximately 50% of gynecologists from UAE- hospitals indicate they have insufficient information about UAE for appropriate counseling and 40% doubts the effectiveness of UAE. Furthermore, in the majority of gynecologists some ‘urban myths’ about the effectiveness and side-effects of UAE seem to persevere.

**Conclusion:**

Adding UAE as a treatment option to the national guideline did not change the number of performed UAEs for symptomatic fibroids. It might be useful to develop an option grid in order to offer appropriate, independent counseling and encourage shared decision making.

**Electronic supplementary material:**

The online version of this article (10.1186/s42155-019-0061-5) contains supplementary material, which is available to authorized users.

## Introduction

Approximately 10–30% of fertile women suffer from heavy menstrual bleeding (Liu et al., 2007). In 40% of these women uterine fibroids are present (Istre et al., 2007). In the last few years multiple randomized controlled trials and a Cochrane review have been conducted and demonstrated UAE to be a valid and safe alternative for hysterectomy in patients with symptomatic uterine fibroids (Hehenkamp et al., 2008;Volkers et al., 2007;van der Kooij et al., 2010;de Bruijn et al., 2016;Pinto et al., 2003;Edwards et al., 2007;Gupta et al., 2014). Long-term follow up showed comparable Health-Related Quality of Life (HRQOL) and treatment satisfaction rates after 5 and 10 years of follow-up (van der Kooij et al., 2010;de Bruijn et al., 2016). Also, UAE is less expensive than hysterectomy (Volkers et al., 2008;van der Kooij et al., 2010). Despite these results, UAE seems to be offered infrequently and implementation seems slow (Voogt et al., 2011). A pilot study published in 2011 showed the lack of a guideline in the management of heavy menstrual bleeding to be the main cause of infrequent UAE in the Netherlands (van der Kooij et al., 2011). In response to this finding, a national guideline for gynecologists was published in 2013. Regarding UAE, this guideline specifically recommends informing the patient of the following outcomes 5 years after the initial treatment (UAE versus hysterectomy); 1) an improved quality of life comparable to that of patients who underwent hysterectomy, 2) faster recovery and return to work after UAE compared to hysterectomy, 3) in 75% of patients who underwent successful UAE no hysterectomy was needed (NVOG 2013).

Most hospitals in the Netherlands are non-profit foundations, whereas most healthcare insurers are for-profit organizations (Götze 2010). In general, there are three types of hospitals; 1) university hospitals, 2) teaching hospitals and 3) general hospitals. University hospitals are academic medical centers connected to the medicine faculty of a major university. These university hospitals provide the most complex and specialized healthcare. The teaching hospitals work together with university hospitals (training of nurses, medical interns and residents) and offer a greater variety of specialisms and treatment options compared to general hospitals. General hospitals provide standard healthcare for less specialized problems and refer, if necessary, to more specialized hospitals (foundation. I, 2018). UAE is considered specialized care. It is executed by interventional radiologists and is offered in all academic hospitals, most teaching hospitals and some general hospitals. Insurance companies allow patients to choose where they want to be treated and with the exception of an obligatory deductible excess, all specialized healthcare costs are covered by the insurer. In these different hospitals standard practice for counseling for UAE differs. Some gynecologists working in UAE+ hospitals include the interventional radiologists in the counseling process, whereas others counsel patients themselves. There is no national guideline of protocol for UAE specifically.

We aimed to evaluate the implementation of UAE in the Netherlands and investigate influential factors concerning gynecologists preference, counseling differences and knowledge.

## Materials and methods

### Study aim, design and setting

The aim of this study was 1) to examine the UAE/hysterectomy ratios before and after introduction of the 2013 Dutch national gynecological guideline for heavy menstrual bleeding, 2) to evaluate the different aspects of UAE counseling, preference, difficulties, knowledge/awareness and suggestions to improve implementation by the use of a questionnaire survey among gynecologists working in UAE+ and UAE- hospitals.

This is a group comparison design study. It was set up in the Dutch health care system and executed among gynecologists working in the Dutch UAE+ and UAE- hospitals.

This study was approved by the Ethics Committee of the coordinating hospital.

### Data collection process

We contacted and asked all 82 Dutch UAE performing and non-UAE performing hospitals to send their annual reports for the extraction of performed hysterectomy numbers. Information about the number of UAEs performed in the consecutive years 2012, 2013 and 2014 were requested from the interventional radiology department.

The questionnaires were sent to selected gynecologists working at hospitals from which we received the annual data and who were identified to be performing hysterectomies themselves. The questionnaire was web based and the link was emailed to the participating gynecologists. In the questionnaire various determinants were examined concerning UAE preferences, counseling content, imaging, implementation and possible improvement suggestions for implementation (Additional file 1).

### Data analysis

Statistical analyses were performed using SPSS statistical software (version 20.0). Results were presented using descriptive statistics such as frequencies, mean (± standard deviation) and median (with interquartile range) as appropriate. In normally distributed and not normally distributed data, respectively the independent T-test and the Mann-Whitney U test were used. In case of categorical data a Chi-square test/Fisher’s exact test was used. *P*-values <.05 were considered statistically significant.

## Results

We received the annual hospital year reports over 2012, 2013 and 2014 of 29/30 (91%) UAE performing and 36/52 (69%) non-UAE performing hospitals. Hysterectomies were documented by detailed operation mode, as mentioned in the annual report. No specific information or ICD-10 codes concerning the indication of the hysterectomy were available. Hysterectomies with vaginoplasty and radical hysterectomies were excluded.

Out of the 30 UAE+ hospitals, we received numbers of performed UAEs from 29/30 radiology departments (91%). The calculated UAE/hysterectomy ratios showed UAE percentages of (9/(120 + 9)) 7,0%, (9/(120 + 9)) 7.0% and (8/(108 + 8)) 6.9% in 2012, 2013 and 2014, respectively. Table 1 outlines the total amount of UAE, hysterectomies in UAE+ hospitals and UAE ratios.

**Table 1:** Mean number of UAE’s, hysterectomies per UAE+ hospital (*n* = 29) and UAE/hysterectomy ratio

Year	Annual report information		
	UAE (SD, range)	Hysterectomies UAE+ hospitals *n* = 29 (SD, range)	UAE∕hysterectomies performed in UAE+ hospitals (%)
2012	9 (19.1; 0–100)	120 (44; 26–212)	7.0
2013	9 (19.1; 0–99)	120 (39; 27–204)	7.0
2014	8 (17.1; 0–91)	108 (51; 0–209)	6.9

When sending the 2017 questionnaires to the hospitals that responded with their annual data, it became apparent that some hospitals started or stopped UAE. The questionnaires were filled in by 43 gynecologists working at UAE+ and 43 gynecologists from UAE- hospitals, resulting in a comparable hospital response rate of 88% (28/32) and 91% (30/33) respectively.

### Counseling and contra-indications for UAE

Gynecologists working at UAE + hospitals estimated to counsel as often (scale 0–10, with 0 meaning “I never counsel” and 10 “I always counsel”: median = 9; IQR 6–10) as gynecologists working at UAE- hospitals (median 7; IQR 3–8), (*p* = .001). Moreover, gynecologists from UAE+ hospitals estimated to counsel a median of 20 patients per year (IQR 1–40), whereas gynecologists in UAE- hospitals counseled a median of 10 patients per year (IQR 1–20) (*p* = 0.021).

Gynecologists were asked to rate contraindications to UAE. Table 2 outlines the differences concerning these ratings between the gynecologists working at UAE- and UAE+ hospitals. None of the ratings were statistically significant different between groups and both groups considered type 7 subserosal pedunculated fibroids and a wish to conceive a contraindication (> 5 on a scale of 0–10).

**Table 2:** Possible contraindications to UAE

Contraindication (Median, IQR)	UAE+ (*n* = 43) (median, IQR)	UAE- (*n* = 43) (median, IQR)	*P*-value
Subserosal pedunculated fibroid (type 7)	7.00 (5–9)	7.00 (5–9)	0.07
Submucous fibroid (type 2)	2.00 (0–5)	1.00 (0–3)	0.68
Only bulky complaints	3.00 (1–7)	3.00 (1–7)	0.71
Suspected concurrent adenomyosis	4.00 (1–7)	5.00 (2–7)	0.15
Uterus size > 20 weeks of gestation	4.00 (2–6)	5.00 (3–8)	0.49
A wish to conceive	9.00 (8–10)	8.00 (8–10)	0.16

Median on a scale of 0–10; 0 = no, it is no contraindication for UAE 10 = yes, I it is a contraindication for UAE

*IQR* Interquartile range *P*-value calculated with Mann-Whitney U test

### Counseling by the interventional radiologist (IR)

Gynecologists working at UAE+ hospitals were asked if the IR is involved in the counseling process. In 18/28 (64%) of UAE+ hospitals the IR is involved. In the remaining 10 UAE+ hospitals the IR is not involved in the counseling process, however 8/14 (57%) of gynecologists working at these hospitals answered this should be standard practice.

In UAE- hospitals 11/43 (26%) of gynecologists stated the IR should not be involved in the counseling process, 12/43 (28%) answered “only if it is preference of the patient” and 16/43 (37%) answered “the interventional radiologist should always be involved”.

### Performing hysterectomies

Almost all (98%) of gynecologist working in UAE+ and UAE- hospital answered they perform hysterectomies. 36/42 (86%) of the gynecologists working at the UAE+ hospitals answered they perform hysterectomies < 50 times a year compared to 31/41 (75%) of the gynecologists at UAE- hospitals. The remaining gynecologists answered they perform 50–100 hysterectomies a year.

### Changes after the 2013 guideline and UAE implementation

Gynecologists answered that UAE is offered more often 16/43 (37%) in UAE+ hospitals versus 19/43 (44%) in UAE- hospitals. In both groups, about half of the group replied nothing changed after the introduced guideline in 2013.Table 3 displays the most interesting distribution of answers to a variety of implementation and counseling questions between the two groups.

**Table 3:** Questions asked in the survey for both gynecologists working at UAE+ and UAE- hospitals

Questionnaire statements	Gynecologists in UAE+ hospitals (%)	Gynecologists in UAE- hospitals (%)	*P*-value
Implementation	Answered yes	Answered yes	
Everybody with fibroids should be counseled for UAE.	98% (42/43)	88% (38/43)	0.20+
I have doubts about the effectiveness of UAE.	0% (0/43)	40% (17/43)	0.00*
I do not wish to transfer care to another specialist.	0% (0/43)	20% (9/43)	0.00+
Counseling	Confirmed statement	Confirmed statement	
1.UAE causes more pain than other treatments.	51% (20/39)	74% (28/38)	0.04*
2. UAE patients recover faster than hysterectomy patients and resume work faster. (true)	82% (32/39)	63% (24/38)	0.06*
3. After a successful UAE, there is a 50% chance of secondary hysterectomy. (false)	46% (18/39)	87% (33/38)	0.00*
Counseling problems and difficulties	Confirmed statement	Confirmed statement	
Option A: The patient does not choose to undergo UAE.	51% (22/43)	51% (22/43)	1.00*
Option B: Logistics are too complicated.	21% (9/43)	NA	
Option C: I do not wish to refer my patient	NA	5% (2/43)	
Option D: It is too complicated to refer	NA	7% (3/43)	
Counseling information	Confirmed statement	Confirmed statement	
1.There is insufficient information available	23% (9/39)	47% (18/38)	0.03*
2. I have sufficient knowledge	77% (30/39)	53% (20/38)	0.03*

*P-value calculated with chi-square and Fisher’s exact (+) test

### Pain management and counseling contents

Regarding pain management, 25/28 (89%) of UAE+ hospitals has a pain protocol containing PCA analgesia in 70% and EDA in 53%.

As displayed in Table 3, the statement “after a successful UAE there is a 50% chance for hysterectomy re-intervention” (*p* = .000) was statistically significant different, between the UAE+ and UAE- hospitals. This statement is false.

Another statistically significant difference (*p* = .03) was observed in favor of UAE+ hospitals concerning knowledge about UAE and counseling. Approximately half of gynecologists working at UAE- hospitals answered they have insufficient information and knowledge for appropriate counseling and 40% of gynecologists in UAE- hospitals doubt the effectiveness of UAE (*p* = 0.00).

### Improvement of available information

Recommendations regarding improvement of UAE counseling was answered as follows: 1) there should be a nationa general patient information leaflet, 2) there should be a national conference day or course day every year and 3) there should be an UAE guideline for gynecologists published on the website of the national association of gynecologists. Also, an option grid was mentioned.

## Discussion

### Main findings

Despite adding the recommendation to counsel on UAE for symptomatic fibroids to the national guideline on heavy menstrual bleeding, no increment in UAE was observed.

It appears that gynecologists working at UAE+ hospitals estimate to counsel more patients for UAE compared to the their colleagues in UAE- hospitals (*p* = 0.001). Gynecologists in UAE+ and UAE- hospitals said UAE is offered more and counseling increased after the 2013 guideline. However, in both groups, about half of the group reported no change after the introduced guideline in 2013 opposing their earlier answers.

Gynecologists from UAE+ and UAE- hospitals showed comparable opinions concerning contra-indications for UAE and most gynecologists from both hospitals agree that the interventional radiologist should be part of the counseling process.

Implementation of UAE in the Netherlands is slow and gynecologists give different answers concerning the cause of this slow implementation.. Concerning UAE counseling, both groups overestimate the risk of re-intervention. This was illustrated by the fact that they indicated the statement “the secondary hysterectomy rate is 50%” to be true. Literature (and also the guideline) state there is a secondary hysterectomy rate of 28% after 5 years of follow up and 31% secondary hysterectomies after 10 years (van der Kooij et al., 2010;de Bruijn et al., 2016). Apparently this has not reached the surveyed gynecologists.

### Interpretations of findings

It is possible that the effect of the 2013 guideline will only become apparent after 2014 since it is known that implementation into general practice may take more time (Grol 1992).

About 51% of gynecologists in UAE+ hospitals versus 52% of the gynecologists in UAE- hospitals mentioned that the patient simply declines UAE when she is counseled. Assuming this is true, why does the patient decline UAE? Twijnstra et al. (Twijnstra et al., 2011) described the discrepancy between preferences of gynecologists for different hysterectomy types owing to variation in daily practice with these types of hysterectomies. He quoted the discrepancy that some countries prevail abdominal hysterectomy as the choice for type of hysterectomy (Garry 2005;Twijnstra et al., 2010;Jacoby et al., 2009) while this is in contrast with the preference of well-counseled patients, who prefer a laparoscopic hysterectomy (Kluivers et al., 2009). It is very likely, that the preference of the patient is influenced by the experience and preference of the counseling gynecologist. Good counseling should be based on facts and should be free of personal preferences of the health care professional. In the case of UAE, counseling is mostly performed by the physician that does not execute the actual procedure. This could be of major influence.

Hysterectomy and UAE are both procedures which could be very painful so a good pain regimen is absolutely necessary. With sufficient pain relief like a PCA pump, patients do have less pain after UAE (Spies 2003). Despite the working frame of the PCA pump, some patients need an alternative pain relief like Epidural Analgesia (EDA) (Hehenkamp et al., 2006). Van der Kooij et al. (van der Kooij et al., 2013) found that EDA would be a more effective pain relief for UAE in the first 6 to 24 h after the UAE. In this study 89% of the UAE+ hospitals said that they had a pain protocol. However, when UAE is considered too painful, there must be inadequate pain relief.

### Strengths and limitations

To our knowledge this is the first study investigating the implementation of UAE in terms of the exact number of performed hysterectomies and UAEs and the counseling process.

We included nearly all UAE+ Dutch hospitals and received a very high response rate of the hospitals and gynecologists, which gave us a valuable reflection of daily practice and thoughts and preferences in the counseling process for UAE.

However, this study is not without limitations. Two hospitals had only the hysterectomies of 2012 and 2013 available and also we had no indications for the hysterectomies since we had the intention to include hysterectomies with indication uterine fibroids. As mentioned earlier, we excluded all hysterectomies with a vaginoplasty but 5 hospitals only mentioned vaginal hysterectomy, so we included these numbers. There could have been some selection bias. The same applies for the hysterectomies with adnexectomy. We included all of these indications (abdominal hysterectomy/vaginal hysterectomy with or without adnexectomy) and maybe there would have been some selection bias, because we did not know exactly how many hysterectomies were performed for the indication of uterine fibroids. However, since this was the same for all inquired years, we do not expect a large bias in the ratio.

The same applies for the UAE’s. Some hospitals fail to describe the indication for UAE and could not discriminate between uterine fibroids and post-partum bleeding. Besides, we observed a large range for the UAEs because 1 hospital was performing 97 UAEs as a mean in the 3 years, not determining for uterine fibroids or post-partum bleeding.

The answers of the gynecologists to the questions were estimates and therefore subjective and exact numbers are lacking. As already mentioned, some hospitals had more gynecologists answering the questionnaire. Nevertheless, it is a good reflection of the daily clinic, since gynecologists in one hospital think different about counseling for UAE. When answers to questions were descriptive, we included all questionnaires. When questions were descriptive, hospitals where more gynecologists answered had a greater part in the overall calculation. This could have been some selection bias, since preferences of gynecologists might cohere with the hospital they are working at. Evaluation of hysterectomy and UAE procedures in 2018 will provide a 5-year follow-up and more insight in implementation.

## Conclusion and recommendations

In conclusion, the publication of the 2013 national guideline which recommends UAE as a treatment option for patients with heavy menstrual bleeding and fibroids did not increase numbers of performed UAEs. The UAE/hysterectomy ratio in 2014 was 6.9, which is unacceptably low for a procedure with solid scientific level 1 evidence.

The availability of UAE influences the frequency and content of counseling in patients with symptomatic uterine fibroids. UAE+ hospitals estimate higher counseling numbers compared to the UAE- hospitals, without apparent influence on UAE numbers.

Despite mentioning facts and fiction on UAE in the national guideline, some ‘urban embolization myths’ tend to persist. Forty percent of gynecologists in UAE- hospitals doubt effectiveness of UAE and nearly half of gynecologists in UAE+ hospitals overestimate the chance of a surgical intervention after UAE. Although there is no scientific evidence from other European countries, oral communications at scientific meetings, seems to support our findings. Since counseling is mostly performed by the physician that does not execute the actual procedure and could therefore influence the patients choice, it might be useful to develop an option grid or decision making tool in order to offer independent counseling and encourage shared decision making, Another option for the physician to become more engaged and exercise a better duty of care role might be a standard multi-disciplinary consultation for patients with fibroids.

## Additional files

Additional file 1: Questionnaires sent to gynecologists working in UAE+ and UAE- hospitals.. (DOCX 18 kb)

## Data Availability

The datasets used and/or analysed during the current study are available from the corresponding author on reasonable request.

## Abbreviations

HRQOL: Health-Related Quality of Life
IQR: Interquartile range
IR: Interventional radiologist
UAE- hospital: Non- Embolization hospital
UAE: Uterine artery embolization
UAE+ hospital: Embolization hospital
