# Supplementary material for: Implementation of uterine artery embolization for symptomatic fibroids in the Netherlands: an inventory and preference study
Source: CVIR Endovasc. 2019 Jun 3;2:18. doi: 10.1186/s42155-019-0061-5 (PMC6966393; doi:10.1186/s42155-019-0061-5)
Supplement: Supplementary file 1 — Questionnaires sent to gynecologists working in UAE+ and UAE- hospitals.. (DOCX 18 kb) [file 42155_2019_61_MOESM1_ESM.docx]

**ADDITIONAL FILE 1. questionnaires for gynecologists working in UAE+ and UAE- hospitals.**

**UAE+ hospital questionnaire.**

1. Do you counsel patients who are eligible for UAE?
2. (never)---------------------------------------------------------------------------10 (always)
3. If the answer is yes, please estimate the total number of patients you have

counseled yearly.

0--------------------------------------------------------------------------------------100

1. Do you refer patients who are eligible for UAE to the interventional radiologist?

0(yes)------------------------------------------------------------------------------10 (no)

1. If the answer is yes, please estimate the total number of patients you have

referred yearly.

0--------------------------------------------------------------------------------------100

1. Would you consider the following characteristics a contra-indication for UAE?

0(yes)------------------------------------------------------------------------------10 (no)

- 1. Type 7 subserosal pedunculated fibroid
  2. Type 2 submucosal fibroid not eligible for trans cervical resection.
  3. Patients with dysmenorrhea, but absence of heavy menstrual bleeding
  4. Patients with suspected concurrent adenomyosis
  5. Patients with an uterus > 20 weeks of gestation
  6. Patients with a wish to conceive

1. Are there any other factors you consider a contra-indication for counseling patients for UAE?
2. Are patients who are scheduled for a UAE also scheduled for an introductory meeting with their treating physician (Interventional radiologist)? Yes/no
3. Does your hospital have a pain protocol for patients undergoing UAE? Yes/no
   1. if the answer is yes, what is the content of this protocol? – Oral medication (NSAID, Paracetamol)– patients controlled analgesia pump – Epidural –other
4. Do you perform hysterectomies? Yes/no
   1. If yes, please estimate how many you perform yearly?
5. Since 2013 the National guideline heavy menstrual bleeding was updated and included UAE as a treatment option in patients with symptomatic fibroids. Following publication of this guideline, did any changes occur in your hospital? If yes, please illustrate with an example.
6. Do you think that (every patient eligible for UAE) everybody should be counseled for UAE? Yes/No
7. Do you have doubts about the effectiveness of UAE? Yes/No
8. Do you wish to transfer care to another spcialist if this is necessary for the treatment?
9. Which items do you discuss during counseling for UAE? (choosing more options is possible)

10.1 UAE causes more pain after treatment than other treatments.

10.2 UAE patients recover faster than hysterectomy patients and resume work faster.

10.3 After a successful UAE, there is a 50% chance of secondary hysterectomy.

10.4 Hysterectomy and UAE offers comparable improvement of health related quality of life and patients satisfaction.

12. Do you have sufficient knowledge concerning the UAE procedure and outcomes to

appropriately counsel your patients? Yes/no

12.1 If no, please offer possible improvement suggestions as how to improve UAE

knowledge and implementation.

1. Which problems do you encounter in daily practice when counseling or planning an UAE?

(choosing more options is possible)

13.1 I do not counsel for UAE

13.2 The patient does not choose to have UAE.

13.3 I do not wish to refer my patient

13.4 Logistics are too complicated.

13.5 UAE is too painful

13.6 Other

**UAE- hospital questionnaire.**

1. Do you counsel patients who are eligible for UAE?

(never)---------------------------------------------------------------------------10 (always)

- 1. If the answer is yes, please estimate the total number of patients you have

counseled yearly.

0--------------------------------------------------------------------------------------100

1. Do you refer patients who are eligible for UAE to a UAE+ hospital?

0(yes)------------------------------------------------------------------------------10 (no)

- 1. If the answer is yes, please estimate the total number of patients you have

referred yearly.

0--------------------------------------------------------------------------------------100

1. Would you consider the following characteristics a contraindication for UAE?

0(yes)------------------------------------------------------------------------------10 (no)

- 1. Type 7 subserosal pedunculated fibroid
  2. Type 2 submucosal fibroid not eligible for trans cervical resection.
  3. Patients with dysmenorrhea, but absence of heavy menstrual bleeding
  4. Patients with suspected concurrent adenomyosis
  5. Patients with an uterus > 20 weeks of gestation
  6. Patients with a wish to conceive

1. Are there any other factors you consider a contra-indication for counseling patients for UAE?
2. Do you think an interventional radiologists should be involved in the counseling process? Yes/No
3. Do you perform hysterectomies? Yes/no
   1. If yes, please estimate how many you perform yearly?
4. Since 2013 the National guideline heavy menstrual bleeding was updated and included UAE as a treatment option in patients with symptomatic fibroids. Following publication of this guideline, did any changes occur in your hospital? If yes, please illustrate with an example.
5. Do you think that (every patient eligible for UAE) everybody should be counseled for UAE? Yes/No
6. Do you have doubts about the effectiveness of UAE? Yes/No
7. Do you wish to transfer care to another specialist if this is necessary for the treatment? Yes/No
8. Which items do you discuss during counseling for UAE? (choosing more options is possible)

10.1 UAE causes more pain after treatment than other treatments.

10.2 UAE patients recover faster than hysterectomy patients and resume work faster.

10.3 After a successful UAE, there is a 50% chance of secondary hysterectomy.

10.4 Hysterectomy and UAE offers comparable improvement of health related quality of life and patients satisfaction.

12. Do you have sufficient knowledge concerning the UAE procedure and outcomes to

appropriately counsel your patients? Yes/no

12.1 If no, please offer possible improvement suggestions as how to improve UAE

knowledge and implementation.

13. Which problems do you encounter in daily practice when counseling or planning an UAE? (choosing more options is possible)

13.1 I do not counsel for UAE

13.2 The patient does not choose to have UAE.

13.3 I do not wish to refer my patient

13.4 Logistics are too complicated.

13.5 UAE is too painful

13.6 Other
